# Pathological findings in a patient with non-dystrophic myotonia with a mutation of the *SCN4A* gene; a case report

**DOI:** 10.1186/s12883-019-1360-0

**Published:** 2019-06-12

**Authors:** Takanori Hata, Takamura Nagasaka, Kishin Koh, Mai Tsuchiya, Yuta Ichinose, Haitian Nan, Kazumasa Shindo, Yoshihisa Takiyama

**Affiliations:** Department of Neurology, Faculty of Medicine, University of Yamanashi, 1110, Shimokato, Chuou-city, Yamanashi, 409-3898 Japan

**Keywords:** Non-dystrophic myotonia, Myotonic dystrophy, Muscle biopsy, *SCN4A*

## Abstract

**Background:**

Non-dystrophic myotonias (NDMs) are skeletal muscle disorders involving myotonia distinct from myotonic dystrophy. It has been reported that the muscle pathology is usually normal or comprises mild myopathic changes in NDMs. We describe various pathological findings mimicking those of myotonic dystrophy (DM) in biopsied muscle specimens from a patient with NDMs with a long disease duration.

**Case presentation:**

A 66-year-old Japanease man presented eye closure myotonia, percussion myotonia and grip myotonia together with the warm-up phenomenon and cold aggravation from early childhood. On genetic analysis, a heterozygous mutation of the *SCN4A* gene (c.2065 C > T, p.L689F), with no mutation of the *CLCN1, DMPK,* or *ZNF9/CNBP* gene, was detected. He was diagnosed as having NDMs. A biopsy of the biceps brachii muscle showed increasing fiber size variation, internal nuclei, chained nuclei, necrotic fibers, fiber splitting, endomysial fibrosis, pyknotic nuclear clumps and disorganized intermyofibrillar networks. Sarcoplasmic masses, tubular aggregates and ragged-red fibers were absent.

**Conclusion:**

It is noteworthy that the present study revealed various pathological findings resembling those seen in DM, although the pathology is usually normal or mild in NDMs. The pathological similarities may be due to muscular modification with long-standing myotonia or excessive muscle contraction based on abnormal channel activity.

**Electronic supplementary material:**

The online version of this article (10.1186/s12883-019-1360-0) contains supplementary material, which is available to authorized users.

## Background

Non-dystrophic myotonias (NDMs) are skeletal muscle disorders involving myotonia distinct from myotonic dystrophy caused by a mutation of the *DMPK* or *ZNF9/CNBP* gene. NDMs comprise a heterogeneous group of neuromuscular disorders caused by mutations of skeletal muscle chloride channels (*CLCN1*) and sodium channels (*SCN4A*), and include myotonia congenita, paramyotonia congenital (PMC), potassium-aggravated myotonia or sodium channel myotonia, and hyperkalemic periodic paralysis (HYPP) [1–3]. In 1991, Ptacek et al. [1] reported a linkage of PMC with the *SCN4A* gene, and suggested that PMC and HYPP are allelic disorders.

Meanwhile, myotonic dystrophy type 2 (DM2), a multisystemic disease manifesting cataracts, grip myotonia, proximal limb muscle weakness, tremors, etc., can exhibit an almost pure myotonic phenotype and is difficult to clinically differentiate from NDMs [3]. The *CLCN1* and *SCN4A* mutations have been reported as modifying factors for DM2 [4, 5].

NDMs are diagnosed based on symptoms, electromyography and muscle pathology before categorization based on genetic analysis. It has been reported that the muscle pathology is usually normal or comprises mild myopathic changes in NDMs [6–9].

We describe various pathological findings mimicking those of DM in biopsied muscle specimens from a patient with NDMs with a long disease duration.

## Case presentation

**Fig. 1:**
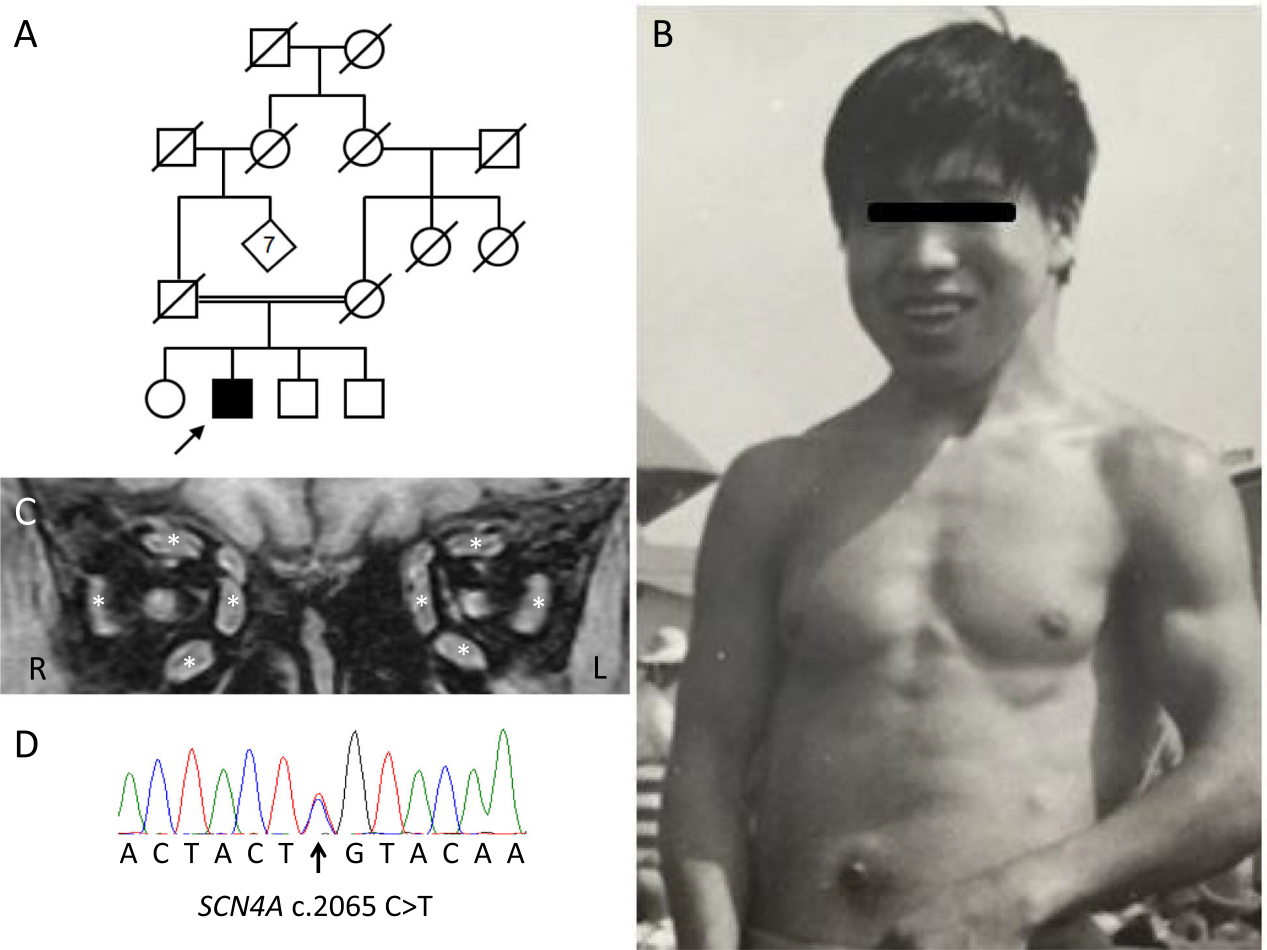
**a** The parents are consanguineous and the family has no neuromuscular disease. **b** Pictures at age of 22. He showed a Hercules-like appearance with hypertrophy of the limbs and axial muscles. **c** Cranial MRI. T1-weighted image. Hypertrophy of all extraocular muscles (asterisks) can be seen. **d** Genetic analysis. A heterozygous missense mutation of the *SCN4A* gene (c.2065 C > T p.L689F) was detected on direct sequencing

A 66-year-old Japanese man complained of increasing muscle stiffness of the four extremities and difficulty in relaxing his grip, which had been present from early childhood. His parents were consanguineous and his family had no neuromuscular disease (Fig. 1a). In his youth, mild muscle weakness and muscular hypertrophy had developed. Later in his life, gait disturbance due to muscle stiffness, severe in the starting period and relieved on continuation of walking, developed. The patient was diagnosed as having myotonia congenita at the age of 22 without any complications (Fig. 1b). He was treated with acetazolamide and phenytoin with relief of the symptoms. In spite of the symptoms, his daily activities were almost normal including working as a gardener, except that his myotonic symptoms got worse at low temperatures. There was no paralytic event during the entire disease course. At the age of 62, his muscular symptoms worsened on discontinuation of phenytoin due to membranous nephropathy, so he was admitted to our hospital. On admission, he had a Hercules-like appearance with hypertrophy of the limbs and axial muscles including the pectoralis major muscles. Physical examination revealed eye closure myotonia, percussion myotonia and grip myotonia. The myotonia improved with muscle exercise or repeated effort, the so-called “warm-up phenomenon”, and was aggravated by exposure to cold. Ocular movement was restricted in every direction with relief on repetition. Dysphagia and dysarthria were also present only when he was exposed to cold. Muscle manual testing was normal at ordinary temperatures.

The other neurologic findings were all normal. There had been no paralytic event or systemic abnormality. Laboratory examination showed an increased level of serum creatine kinase, 388 U/l (normal value < 279 U/l), which decreased with rest in hospital. Electrocardiography, ultrasound cardiography and chest X-raying were normal. Needle electromyography of the left biceps brachii muscle and quadriceps femoris muscle showed myotonic discharges (Additional file 1). T1-weighted images of Cranial MRI revealed hypertrophy of all extraocular muscles (Fig. 1c). His myotonic symptoms improved since the start of administration of mexiletine (300 mg/day).

Genetic analysis: Whole-exome sequencing revealed a heterozygous mutation of the *SCN4A* gene (c.2065 C > T, p.Leu689Phe). However, we found no mutation of the *CLCN1* gene. Then, we confirmed the *SCN4A* mutation by direct sequencing (Fig. 1d). Mutation analysis showed no mutation of the *DMPK* or *ZNF9/CNBP* gene.

### Muscle biopsy

**Fig. 2:**
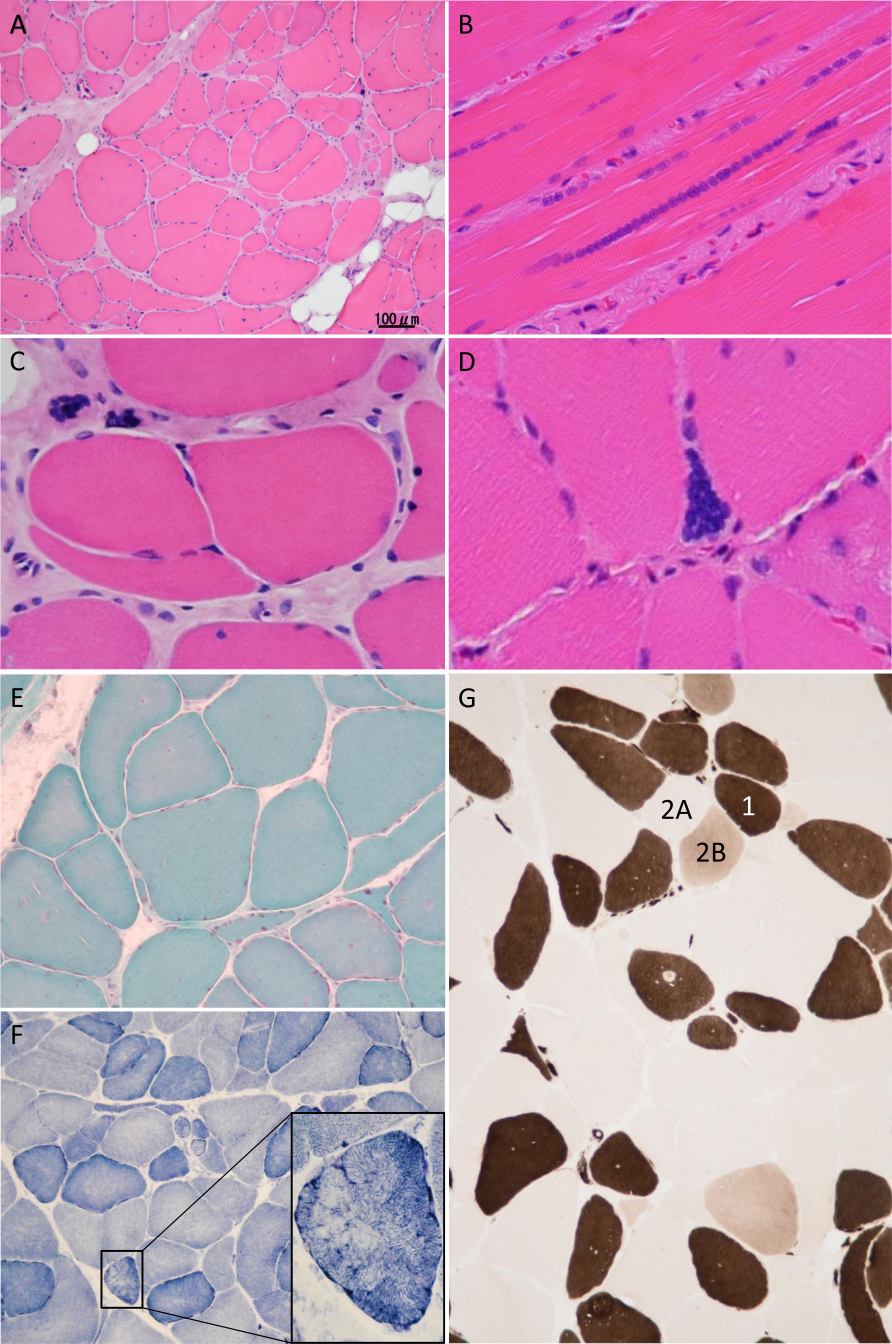
**a**-**d** HE staining. Variation in diameter of muscle fibers including hypertrophy (over 100 μm) and atrophy was observed (**a**). Internal nuclei, chained nuclei (**b**), fiber splitting (**c**), pyknotic nuclear clumps (**d**), endomysial fibrosis and mild fatty replacement were observed in all areas. **e** modified Gomori trichrome staining. Ragged-red fibers could not be observed but fibers exhibiting slight marginal hyperstaining were observed. **f** NADH-TR(β-nicotinamide adenine dinucleotide tetrazolium reductase)staining. Disorganized intermyofibrillar networks including a moth-eaten appearance and lobulated fibers could be seen. **g** Myosin ATPase staining. Reduction of type 2B fibers and predominancy of type 2A fibers could be seen

A muscle biopsy was performed on the right biceps brachii muscle, followed by light microscopy and routine electron microscopy. On HE staining, variation in diameter of muscle fibers including hypertrophy (over 100 μm, about a half of muscle fibers) and atrophy was observed (Fig. 2a, Additional files 2 and 3). Internal nuclei, chained nuclei (Fig. 2B), fiber splitting (Fig. 2c), pyknotic nuclear clumps (Fig. 2d), endomysial fibrosis and mild fatty replacement were observed in all areas. Sarcoplasmic masses were absent. On modified Gomori trichrome staining (mGT), ragged-red fibers could not be observed but fibers exhibiting slight marginal hyperstaining were observed (Fig. 2e). NADH-TR staining showed disorganized intermyofibrillar networks including a moth-eaten appearance and lobulated fibers (Fig. 2f). Myosin ATPase staining showed reduction of type 2B fibers and predominancy of type 2A fibers (Fig. 2g). On electron microscopy, atrophic fibers exhibiting Z-streaming were observed (Additional file 2). Tubular aggregates were absent in all examinations.

## Discussion and conclusion

In the past, NDMs were classified into three genetic categories, i.e., dominant or recessive *CLCN1* mutations, and dominant *SCN4A* ones. However, it has been clarified that myotonias with these mutations have various clinical phenotypes [3, 10–12].

Irrespective of causative channel mutations, the muscle pathologies of NDMs are limited to mild nonspecific myopathic changes [6]. There is no major pathological qualitative difference between mutated *SCN4A* and mutated *CLCN1* muscle. Mild variation of fiber size and type 2 predominancy with a reduction or absence of type 2B fibers were commonly noted in several cases with mutated *SCN4A* [7, 8], and mutated *CLCN1* [9, 13, 14]. Other findings, including centrally located nuclei [8] and excessive mitochondria without aggregation in NDMs [15–17], an increase in endomysium connective tissue in *CLCN1* [18], and tubular aggregates and vacuoles in myotonia congenita [19] have been reported. There were few findings supportive of light microscopic findings as to ultrastructural changes [19, 20]. In the present case, different from the above findings, the muscle pathology closely resembled that in DM, as summarized in Table 1 [21, 22]. Increased internal nuclei, pyknotic nuclear clumps, chained nuclei and fiber splitting are the same findings as in DM. Other findings including variation of fiber diameter, endomysial fibrosis, fiber type predominancy and the type of atrophy differ between DM and our case. In summary, major differences in our case compared with DM are as follows, the dystrophic changes are not so severe, and regeneration of muscle fibers is superior to degeneration. The reason why the pathological changes in NDMs were stated to be mild in the past reports may be that the examinations were performed in childhood or adolescence. As in the present case, pathological changes similar to those in DM might be due to the long term myotonia or excessive muscle contraction, although the mechanism of muscle degeneration is likely not the same for the two diseases.

**Table 1:** Comparison of pathological findings between DM1, DM2 and the present case

	Present case	DM1	DM2	NDMs
Fiber size variation	++	+++	+++	+
Endomysial fibrosis	+	+++	++	±
Increase of internal nuclei	+++	+++	+++	±
Pyknotic nuclear clumps	+	+	+++	–
Sarcoplasmic masses	–	++	±	–
Chained nuclei	+	+	unknown	–
Fiber splitting	+	+	unknown	–
Ragged red fibers	–	+	±	–
Fiber type predominancy	2A > 1> > 2B	type1	type1	2A > 1> > 2B
Type of atrophy	nonspecific	type1 > type2	type2	type2B
Ring fibers	–	++	±	–
Fatty replacement	+	+++	++	–

+++ severe; ++ modest; + mild; ±occasionally present; − absent

Schoser et al. [20] proposed that sodium channelopathies may mimic the phenotypic features of DM2 in older patients based on the muscle pathology in patients with a heterozygous mutation of the *SCN4A* gene (A1481D). Although the pathological features of NDMs are essentially different from DM1 and DM2 [21], see Table 1., DM2 is clinically the most important disorder for differential diagnosis of NDMs in adulthood. It is because DM2 has mild muscular involvement and mild or few systemic complications [23]. So we analyzed *DMPK* and *CNBP* for differential diagnosis, and confirmed no mutation.

Genetic screening of *SCN4A* in our case revealed a heterozygous missense mutation (c.2065C > T, p.Leu689Phe). The mutation was reported only in one family i.e., the proband and her daughter, as a de novo mutation [24]. The proband of the family showed a Thomsen-like phenotype without muscle weakness like in the present case.

In daily practice, the diagnosis of NDMs or DM is based on clinical findings and on genetic testing, and a muscle biopsy in the patients is dispensable. A muscle biopsy in patients with myotonia is significant in terms of a better understanding of the pathophysiology. We need further examination for associating the described pathological findings with *SCN4A*-related NDMs.

## Additional files

Additional file 1: **Figure S1.** Needle electromyography. Myotonic discharges could be seen for the left biceps brachii muscle and quadriceps femoris. (PPTX 194 kb)

Additional file 2: **Figure S2.** Electron microscopic findings. Atrophic fibers exhibiting Z-streaming were observed. (PPTX 321 kb)

Additional file 3: **Figure S3.** Diameter of muscle fibers. Muscle fiber diameter was determined for 234 fibers in cross-section. The average diameter is 100.6 μm, ranging from 6.8 to 118.2. 48.7% of muscle fibers exhibit hypertrophy (over 100 μm). (PPTX 46 kb)

## Data Availability

All data generated or analysed during this study are included in this published article.

## Abbreviations

ATPase: Adenosine triphosphatase
DM: Myotonic dystrophy
HE: Hematoxylin-eosin
HYPP: Hyperkalemic periodic paralysis
mGT: Modified Gomori trichrome staining
MRI: Magnetic resonance imaging
NADH-TR: β-nicotinamide adenine dinucleotide tetrazolium reductase
NDMs: Non-dystrophic myotonias
PMC: Paramyotonia congenital
