# Supplementary figures and images for: Pathological findings in a patient with non-dystrophic myotonia with a mutation of the SCN4A gene; a case report
Source: BMC Neurol. 2019 Jun 12;19:125. doi: 10.1186/s12883-019-1360-0 (PMC6560775; doi:10.1186/s12883-019-1360-0)

## Slide 1
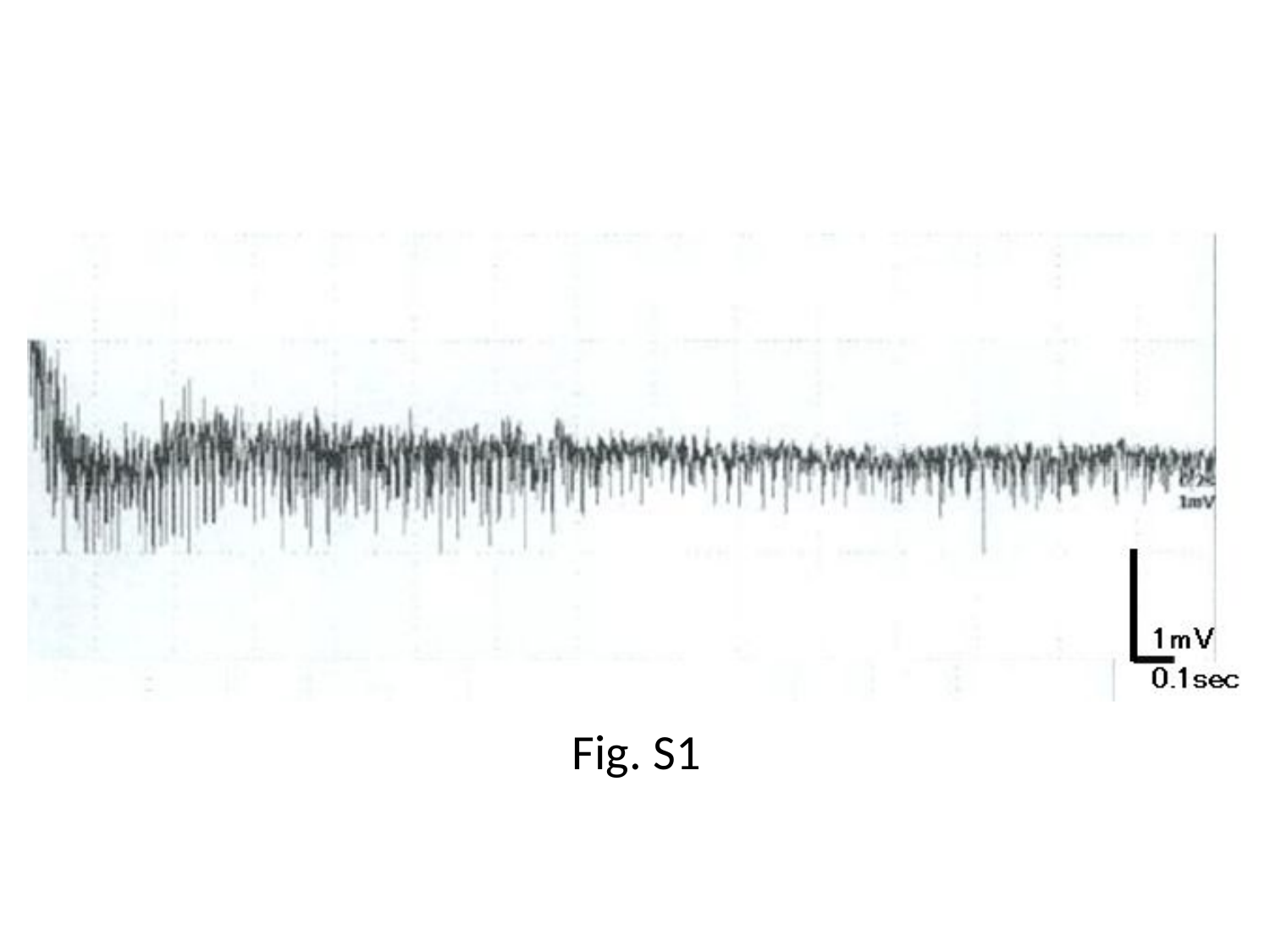

Fig. S1

Supplement: Supplementary file 1 — Figure S1. Needle electromyography. Myotonic discharges could be seen for the left biceps brachii muscle and quadriceps femoris. (PPTX 194 kb) [file 12883_2019_1360_MOESM1_ESM.pptx]

## Slide 1
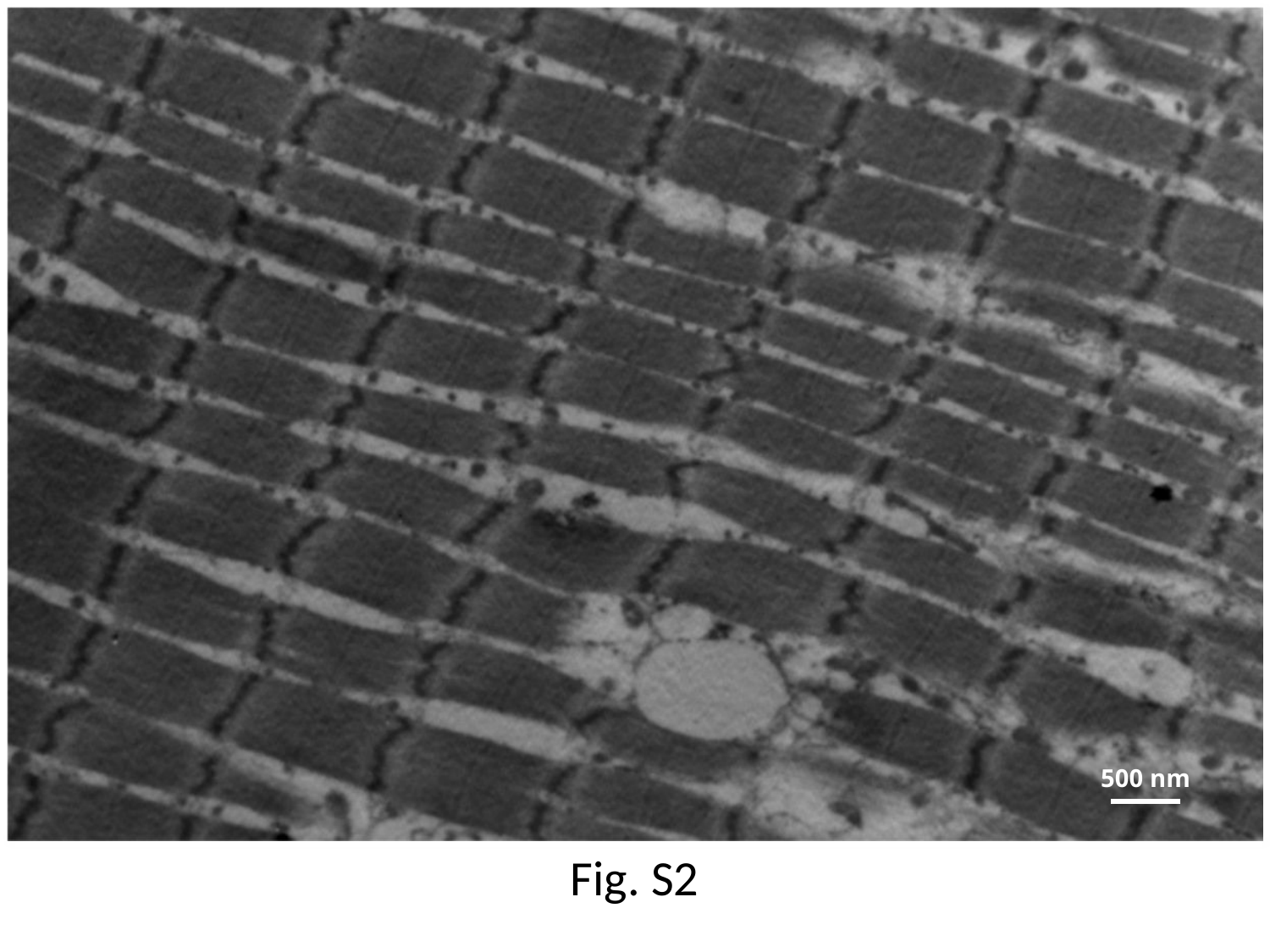

500 nm
Fig. S2

Supplement: Supplementary file 2 — Figure S2. Electron microscopic findings. Atrophic fibers exhibiting Z-streaming were observed. (PPTX 321 kb) [file 12883_2019_1360_MOESM2_ESM.pptx]

## Slide 1
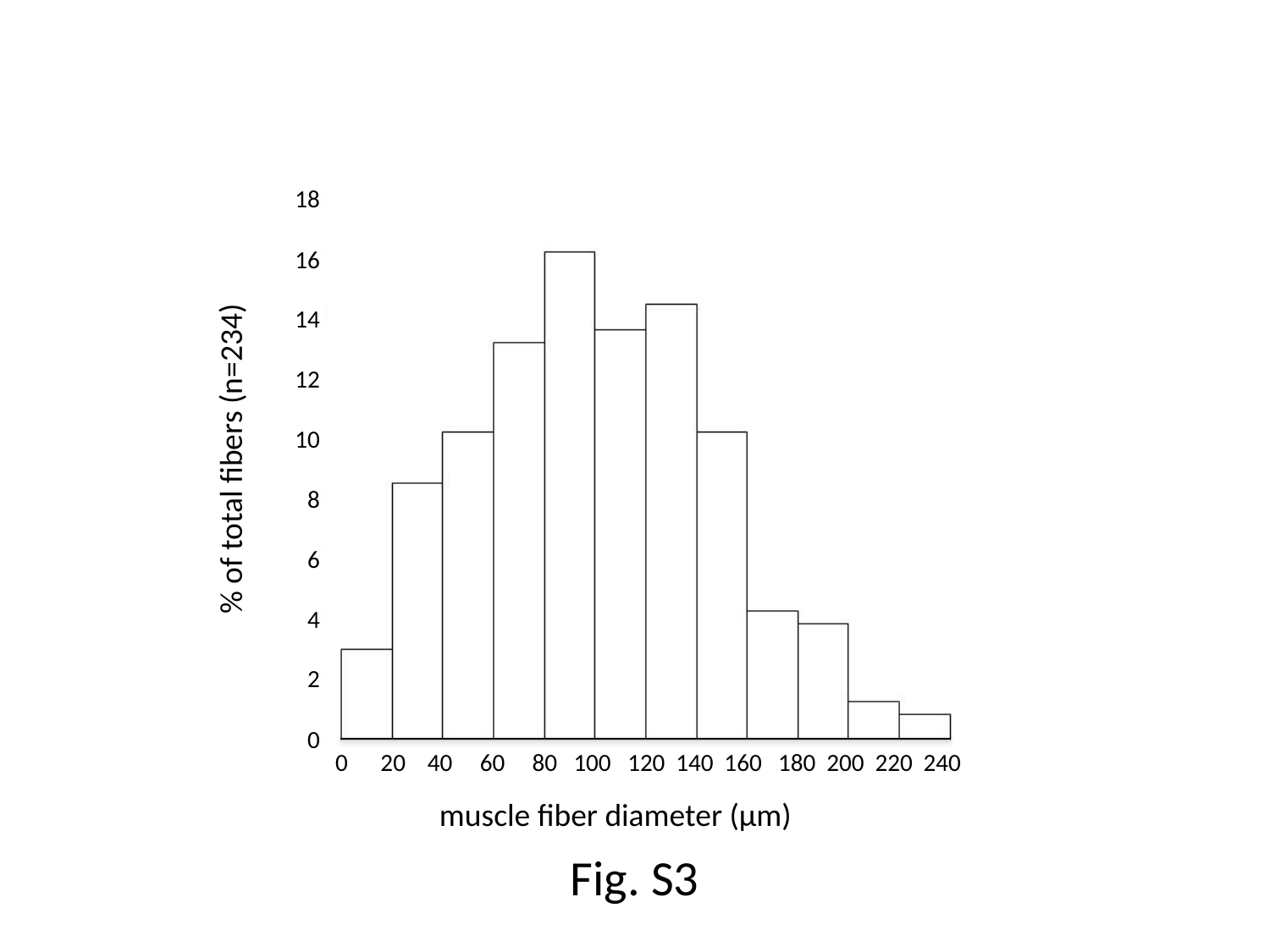

18
16
14
12
10
% of total fibers (n=234)
8
6
4
2
0
0 20 40 60 80 100 120 140 160 180 200 220 240
muscle fiber diameter (μm)
Fig. S3

Supplement: Supplementary file 3 — Figure S3. Diameter of muscle fibers. Muscle fiber diameter was determined for 234 fibers in cross-section. The average diameter is 100.6 μm, ranging from 6.8 to 118.2. 48.7% of muscle fibers exhibit hypertrophy (over 100 μm). (PPTX 46 kb) [file 12883_2019_1360_MOESM3_ESM.pptx]
